# Unravelling the pharmacological properties of cryptolepine and its derivatives: a mini-review insight

**DOI:** 10.1007/s00210-022-02302-7

**Published:** 2022-10-17

**Authors:** Champa Keeya Tudu, Anustup Bandyopadhyay, Manoj Kumar, Radha, Tuyelee Das, Samapika Nandy, Mimosa Ghorai, Abilash Valsala Gopalakrishnan, Jarosław Proćków, Abhijit Dey

**Affiliations:** 1 Department of Life Sciences, Presidency University, 86/1 College Street, Kolkata, -700073 West Bengal India; 2 Chemical and Biochemical Processing Division, ICAR-Central Institute for Research On Cotton Technology, Mumbai, 400019 Maharashtra India; 3 School of Biological and Environmental Sciences, Shoolini University of Biotechnology and Management Sciences, Solan, 173229 Himachal Pradesh India; 4 Department of Biomedical Sciences, School of Biosciences and Technology, Vellore Institute of Technology (VIT), Vellore, Tamil Nadu 632014 India; 5 Department of Plant Biology, Institute of Environmental Biology, Wrocław University of Environmental and Life Sciences, Kożuchowska 5b, 51-631 Wrocław, Poland

**Keywords:** Cryptolepine, *Cryptolepis sanguinolenta*, Antitumor, Anti-inflammatory, Antimalarial, Hepatoprotective, Pharmacology

## Abstract

Cryptolepine (1,5-methyl-10*H*-indolo[3,2-*b*]quinoline), an indoloquinoline alkaloid, found in the roots of *Cryptolepis sanguinolenta* (Lindl.) Schltr (family: Periplocaceae), is associated with the suppression of cancer and protozoal infections. Cryptolepine also exhibits anti-bacterial, anti-fungal, anti-hyperglycemic, antidiabetic, anti-inflammatory, anti-hypotensive, antipyretic, and antimuscarinic properties. This review of the latest research data can be exploited to create a basis for the discovery of new cryptolepine-based drugs and their analogues in the near future. PubMed, Scopus, and Google Scholar databases were searched to select and collect data from the existing literature on cryptolepine and their pharmacological properties. Several in vitro studies have demonstrated the potential of cryptolepine A as an anticancer and antimalarial molecule, which is achieved through inhibiting DNA synthesis and topoisomerase II. This review summarizes the recent developments of cryptolepine pharmacological properties and functional mechanisms, providing information for future research on this natural product.

## Introduction

**Fig. 1:**
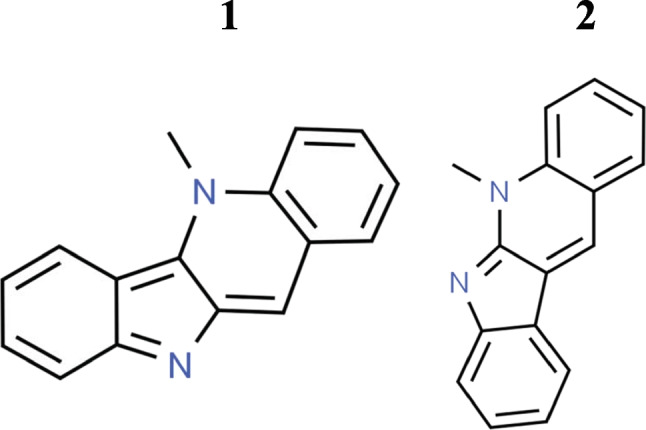
Chemical structure of 1 cryptolepine and 2 neocryptolepine/cryptotackieine

**Fig. 2:**
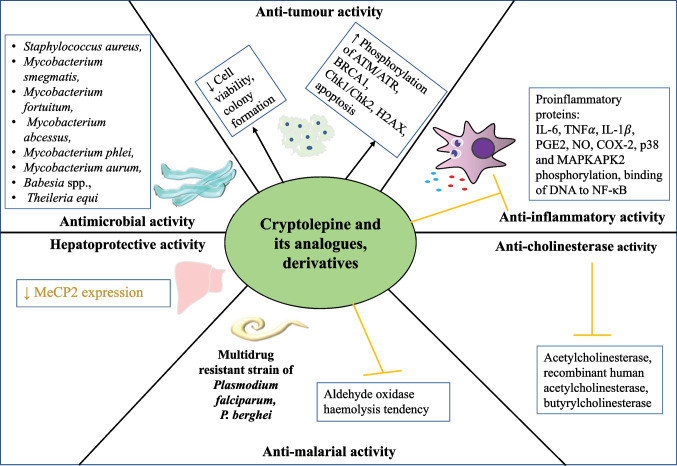
Diagram with the relevant pharmacological properties of cryptolepine and its analogues, derivatives, and their potential mechanism of actions

Cryptolepine (1,5-methyl-10*H*-indolo[3,2-*b*]quinoline) (Fig. 1; Table 1) is an indoloquinoline alkaloid isolated from the roots of scrambling shrub *Cryptolepis sanguinolenta* (Lindl.) Schltr (family: Periplocaceae), which is found in Central and West Africa (Seville et al. 2007). The aqueous root extracts from this plant have been traditionally used for the treatment of malaria, rheumatism, urinary tract infections, upper respiratory tract infections, and intestinal disorders in mainly Central and West African countries such as Ghana and Nigeria (Pal and Katiyar 2016). *C. sanguinolenta* was approved by the Food and Drugs Authority in Ghana to perform clinical trials for the treatment of COVID-19. Cryptolepine has been reported to possess various pharmacological properties, including antimalarial, anti-bacterial, anti-fungal, anti-hyperglycemic, anticancer, antidiabetic, anti-inflammatory, hypotensive, and antipyretic properties (Wright 2007; Forkuo et al. 2016; Mensah et al. 2019; Batiha et al. 2020; Shnyder and Wright 2021; Abacha et al. 2022). Cryptolepine is used as an antimalarial in Central and West African countries. The root decoction of *C. sanguinolenta* is used in conventional medicine for the treatment of malaria as well as for infectious and non-infectious diseases. Antimalarial properties of cryptolepine were studied in vitro. In addition to treating malaria, it also helps with colic and stomach ulcers. Besides that, it also has anticancer properties through inhibition of DNA synthesis or inhibition of topoisomerase II. Interestingly, it stimulates the cleavage of DNA at a subset of the pre-existing topoisomerase II cleavage sites and topoisomerase II-DNA covalent complexes (Lisgarten et al. 2002). *C. sanguinolenta* root extract is used in herbal formulations in orthodox Ghanaian clinics. The plant is widely accepted for treating malaria or using correlative treatments when standard antimalarial medicine is unavailable since the plant has a high correlation with the safety and efficacy of a teabag formulation (Phyto-laria) against chloroquine-resistant strains of *Plasmodium falciparum* in human clinical experiments (Forkuo et al. 2017a). There have also been many researchers who have found that cryptolepine is not an optimal antimalarial drug because of its toxicity; however, there have been many laboratories that have synthesized derivatives of cryptolepine to find properties that have decreased cytotoxic/DNA interactions and more effective antiplasmodial activities. Various studies have also concluded that cryptolepine has the potential to inhibit topoisomerase II and has the potential to be a useful antitumor medicine. It stimulates the cutting of DNA at a subset of pre-existing topoisomerase II cleavage sites and topoisomerase II-DNA covalent complexes (Lisgarten et al. 2002). Furthermore, cryptolepine has been found to have cytotoxic properties against mammalian cancers. The purpose of this review is to shed light on the possible roles and biological properties of cryptolepine. Researchers are attempting to provide a concise, up-to-date summary of the pharmacological activity and purpose of cryptolepine (Fig. 2). This review aims at documenting the latest developments in pharmacological properties and functional mechanisms of cryptolepine and providing useful data for future development and exploration of this natural product.

**Table 1:** Properties of phytoconstituents

Compound name	Molecular formula	Average mass (Da)	Average mass (Da)
Cryptolepine	C_16_H_12_N_2_	232.280	232.100052
Neocryptolepine/cryptotackieine	C_16_H_12_N_2_	232.280	232.100052

## Review methodology

A study was conducted on PubMed-NCBI, NISCAIR Online Periodicals Repository (NOPR), SpringerLink, ScienceDirect, Scopus, and Google Scholar using various combinations and their synonyms: “Cryptolepine,” “*Cryptolepis sanguinolenta*,” “in vitro,” “anti-inflammatory,” “antimicrobial,” “anti-protozoal,” “antitumor,” “antidiabetic,” and “antimalarial.”Many data were recaptured from these websites and some scientific research engines. There have also been references to some traditional documents such as books and manuscripts. The information about pharmacological activities of the alkaloid cryptolepine was provided by cross-referencing results. The therapeutic attributes of cryptolepine have been compiled into different sections each focusing on different bioactivity along with the medicinal properties isolated from *C. sanguinolenta*. This comprehensive review will provide a suitable source for basic and clinical investigations of cryptolepine. Tables 1, 2, 3, and 4 represent the physical properties of constituents, the antitumor or anticancer, antimalarial, and antidiabetic activities of cryptolepine and its analogues and derivatives, respectively.

**Table 2:** Antitumor and anticancer activities of cryptolepine and its analogues and derivatives

Cryptolepine and its analogues and derivatives	Experimental model/ assay	Type of study	Results/mechanisms of action		Reference
C-11 diamino cryptolepine derivatives NSC748392, NSC748393, NSC748394	Mammalian non-tumor cells (Vero cells), drug ability properties	In vitro, FRET-melting assays,	Multi-target mechanism, G-Quadruplex DNA binding affinity, GI_50_ averages at sub-micromolar concentrations (0.32– 0.78 µM)		Lavrado et al. 2010
Cryptolepine	Several solid human tumors with breast tumors	In vitro	Inflammatory and anti-apoptotic genes such as COX-2, iNOS, TNFa, Bcl-2↓; proapoptotic genes such p53, p21, Bax, caspase and cytochrome C↑		Ansha and Mensah 2013
Nitro analogues of cryptolepine such as 2-chloro-7-nitrocryptolepine hydrochloride, 2-chloro-9-nitrocryptolepine hydrochloride, 2-fluoro-7-nitrocryptolepine hydrochloride, 2-fluoro-9-nitrocryptolepine hydrochloride, 8-chloro-7-nitrocryptolepine hydrochloride, 8-chloro-9-nitrocryptolepine hydrochloride, 7-bromo-8-nitrocryptolepine hydrochloride	Non-small cell lung carcinoma cell line (H460), human colon carcinoma cell line (BE), RT112 cell line	In vitro		IC_50_ values < 2 µM, substrates for NQO1 or NQO2, intercalate into DNA at GC rich sequences, topoisomerase II↓	Seville et al. 2007
8-Fluoro-10-(N-3-dimethylaminopropyl)amino-11H-indeno[1,2-b]quino- -line	Human cancer cell lines (HepG-2, T24, NCI-H460 and MGC-803) and one normal human cell line (HL-7702)	In vitro		IC_50_ values from 0.31 to 11.97 µM, pro-apoptotic proteins Bak, Bax and Bim ↑, anti-apoptotic proteins Bcl-2 and Bcl-xL ↓, and effector caspase-3/9 were activated to initiate apoptosis	Yuan et al. 2019
Cryptolepine and neocryptolepine	P388 murine leukemia cells, HL-60 human leukemia cells	In vitro		Asp-Glu-Val-Asp- or Ile-Glu-Thr-Asp-caspases↑, topoisomerase II↓	Dassonneville et al. 2000
Zn(II)—cryptolepine-curcumin derivatives, such as [Zn(BQ)Cl2] (BQ-Zn) and [Zn(BQ)(Cur)]Cl (BQCur-Zn)	Human bladder (T-24) tumor cells	In vivo and in vitro		↓MMP loss, ↓ tumor growth in the dark and under light irradiation, TCM metal complexes, ↓mitochondrial dysfunction	Qin et al. 2020
Synthetic cryptolepine as the sulfate salt	12 different human tumor cell lines	In vitro		Highest correlations to topoisomerase II and microtubule targeting drugs	Laryea et al. 2009
Pt(II) compounds with [5-(benzo[4,5]furo[3,2- b]quinolin-11-yloxy)-pentyl]-bis-pyridin-2-ylmethyl-amine (BQL1) and [9-(benzo[4,5]furo[3,2-b]quinolin-11-yloxy)-nonyl]-bispyridin-2-ylmethyl-amine (BQL2)	T-24 cancer cells and normal HL-7702 cells	In vivo and in vitro		Active at micromolar range (1.3 ± 0.1 and 0.2 ± 0.2 μM, respectively), mitochondrial apoptosis pathway, apoptotic proteins	Qin et al. 2021

**Table 3:** Antimalarial activities of cryptolepine and its analogues and derivatives

Cryptolepine and its analogues and derivatives	Source	Method	Type of study	Parasite	Effects	Reference
Cryptolepine derivatives, 2,7-dibromocryptolepine hydrochloride	Synthetic	Cultured K1 strain of *P. falciparum*	In vitro	*Plasmodium falciparum*	IC_50_ = 0.44 ± 0.22 µM	Wright et al. 2001
					IC_50_ = 0.049 ± 0.017 µM	
		*P. berghei* infected mice by injection (i.p.) at a dose of 12.5 mg kg^−1^ day^−1^	In vivo	*Plasmodium berghei*	Suppression of parasitemia 89.1%	
7-bromo-2-chlorocryptolepine hydrochloride, 2-bromo-7-nitrocryptolepine hydrochloride	Synthetic	*P. berghei* infected mice at doses of 25 mg kg^−1^ day^−1^	In vivo	*Plasmodium berghei*	Suppressed parasitemia by > 90%	Onyeibor et al. 2005
Cryptolepine derivatives	Synthetic	Cultured K1 strain of *P. falciparum*	In vitro	*Plasmodium falciparum*	IC_50_ values < 0.1 µM	
Cryptolepine	Natural	Cultured 3D7 strain of *P. falciparum*	In vitro	*Plasmodium falciparum*	IC_50_ = 603.82 ± 75.57 nM	Forkuo et al. 2016
Cryptolepine triflate, 11-(4- piperidinamino) cryptolepine hydrogen dichloride	Synthetic	Chloroquine-resistant, pyrimethamine-resistant and cycloguanil resistant K1 strains of *P. falciparum*	In vitro	*Plasmodium falciparum*	IC_50_ ≤ 1.4 µM	e Silva et al. 2012
Cryptolepine	Natural	Cultured 3D7 strain of the late-stage gametocytes of *P. falciparum* (NF54)	In vitro	*Plasmodium falciparum*	IC_50_ = 1965 nM	Forkuo et al. 2017a
Cryptolepine and its hydrochloride, 11- hydroxycryptolepine, neocryptolepine	Synthetic	Cultured K1 and W2 strains of *P. falciparum*	In vitro	*Plasmodium falciparum*	IC_50_ values of 42 ± 0.1 and 54 ± 0.7 ng/mL	Cimanga et al. 1997

**Table 4:** Antidiabetic activities of cryptolepine and its analogues and derivatives

Cryptolepine and its analogues and derivatives	Experimental model/ method	Type of study	Result and mechanism	Reference
Cryptolepine (10,30, or 100 mg/kg) / natural	Diabetic Sprague–Dawley rats	In vitro	Intracellular calcium in the beta cell ↑, plasma glucose concentration ↓, hyperglycemia ↓, weight ↓, cold allodynia, and neuropathic pain	Ameyaw et al. 2016
Hydroiodide, hydrochloride, and hydrotrifluoromethanesulfonate (hydrotriflate) salts of cryptolepine/synthesized	3T3-L1 glucose transport assay	In vitro	Plasma glucose concentrations↓	Bierer et al. 1998b
	Fructose-Fed STZ-Treated rats	In vivo		
Cryptolepine derivatives/synthesized	Obese diabetic mice (designated C57BL/KS-db/ db or db/db)	In vivo	Plasma glucose concentrations↓, food intake↑	Bierer et al. 1998a

## Pharmacological properties of cryptolepine and its analogues and derivatives

### Antitumor activity

Cryptolepine acts against various types of cancer. In preclinical studies, the main alkaloid of *C. sanguinolenta* cryptolepine showed the greatest cytotoxic activity against several solid human tumors including breast tumors. Cryptolepine has demonstrated clinical activity in breast tumors, specifically affecting cyclins D1, D2, and D3 and cyclin E, which regulate the cell cycle (Ansha and Mensah 2013). The antitumor activity of cryptolepine has also been demonstrated in human non-melanoma skin cancer cells (NMSCCs) (Pal and Katiyar 2016). Using normal human epidermal keratinocytes as a comparison, both SCC-13 and A431 cell lines showed higher levels and activity of topoisomerase (Topo I and Topo II) than normal keratinocytes, and this activity decreased significantly after cryptolepine treatment of NMSCC via the comet assay (Pal and Katiyar 2016). As a result, cryptolepine caused DNA damage, and ATM/ATR, BRCA1, Chk1/Chk2, and H2AX phosphorylation was also increased, and the p53 signaling cascade was activated, and mitochondrial membrane potential was disrupted. Therefore, cryptolepine showed a significant reduction in cell viability, colony formation, and an increase in apoptosis (Pal and Katiyar 2016). Yuan et al. (2019) evaluated the anti-proliferative effects of thirty cryptolepine derivatives on four human tumor cell lines (HepG-2, T24, MGC-803, NCI-H460) as well as one human normal liver cell line (HL-7702). Among the cryptolepine derivatives, one referred to as 8-fluoro-10-(*N*-3-dimethylaminopropyl)amino-11*H*-indeno[1,2-*b*]quinolone could strongly bind to G-quadruplex, since cells in the S/G2 phase is arrested and undergo apoptosis. Additionally, this study showed that cryptolepine exhibited anticancer activity with no obvious toxicity and the tumor growth inhibition (TGI) up to 53.2% in the MGC-803 xenograft tumor model (Yuan et al. 2019). Cryptolepine was investigated for evaluating cytotoxicity in 12 human tumor cell lines and in primary cultures of tumor cells from patients and DNA microarray analysis of cryptolepine to estimate gene expression by the fluorometric microculture cytotoxicity assay. The mean cryptolepine IC_50_ values in this study were0.9 μM compared with 1.0 and 2.8 μM in hematological and solid tumor malignancies, respectively, and correlated with topoisomerase II and microtubule targeting drugs (Laryea et al. 2009). Zhu and Gooderham (2006) investigated the anticancer activity of cryptolepine against p53-dependent and independent molecular events in human lung adenocarcinoma A549 cells, which were associated with cell cycle alteration and cell death. In a 24-h treatment, cryptolepine induced p21^Cip1/WAF1^ aggregation, which had up to 5 mM and 1.25–10 mM p53 concentrations. Cells inflicted with cryptolepine induced G1-phase block, S-phase block, G2/M-phase block, and the dead cells displayed condensed and fragmented nuclei or some apoptosis features. In this study, cryptolepine activated multiple pathways involved in cell cycle arrest at the G1 phase via using a specific inhibitor of DNA-PK and siRNA-mediated p53 silencing (Zhu and Gooderham 2006). Moreover, cryptolepine demonstrated anticancer activity against p53-mutated human osteosarcoma MG63 cells, which activated the p21^*WAF1/CIP1*^ expression with growth arrest as reported by Matsui et al. (2007). Activation of cryptolepine resulted in an arrest of the growth of MG63 cells (Matsui et al. 2007). Results raised the possibility that treatment with cryptolepine is very useful for the chemotherapy of human osteosarcoma. The antitumor and anticancer activities of cryptolepine and its derivatives are listed in Table 2.

### Antimalarial activity

Malaria is an infectious disease caused by the genus *Plasmodium*, transmitted to humans through the bite of infected mosquitoes. In Africa, it leads to one million deaths per year among children under the age of 5 years. Cryptolepine and cryptolepine analogues are used as major components for the development of antimalarial drugs. Cryptolepine has potent activity against both chloroquine-sensitive and chloroquine-resistant *Plasmodium falciparum.* Cryptolepine is sometimes unsuitable as an antimalarial medicine due to its toxicity, but derivatives of cryptolepine can have lower cytotoxicity and are used to cure malaria (Gopalan et al. 2011). Study findings indicate that various derivatives of cryptolepine have the potential for development into antimalarial drugs (Stell et al. 2012). Stell et al. (2012) documented the antimalarial activity of 2-fluorocryptolepine, which was converted to its 11-one analogue by rabbit liver aldehyde oxidase. These analogues block the aldehyde oxidase and stop malarial activity. Lavrado et al. (2011) investigated the antimalarial activities of cryptolepine derivatives consisting of basic side chains at the C-11 position, and the side chains including propyl, butyl, and cycloalkyl diamine significantly increased activity against chloroquine-resistant *P. falciparum* strains (Lavrado et al. 2011). In 2012, Kuntworbe and Al-Kassas published a study describing the cryptolepine hydrochloride-loaded gelatin nanoparticles. Further investigation was conducted into developing formulations that would improve malaria treatment through in vitro hemolytic evaluation. It was concluded that drug-loaded nanoparticles were more likely to reduce hemolysis tendency compared to pre-formed nanoparticles (2.5ad and 11.0ad). These drug-loaded nanoparticles were assayed with erythrocyte suspension, revealing an increased contact between the drug on the nanoparticle surface and erythrocytes (Kuntworbe and Al-Kassas 2012). A good antimalarial drug has a sustained and moderate release of the antimalarial agent from the formulations. In a Sprague–Dawley rat model, Forkuo et al. (2016) reported that a combination of cryptolepine and artemisinin derivatives showed synergistic antimalarial activity in vivo and in vitro against *P. berghei* NK-65 and *P. falciparum* 3D7. During in vitro study, the cryptolepine along with some artemisinin derivatives compared in the SYBR Green I, fluorescent-based, drug sensitivity assay executed on the CQ-sensitive plasmodial strain 3D7, and in vivo cryptolepine estimated a similar decrease in the artesunate-treated groups with a significant dose-dependent reduction in parasitemia levels (Forkuo et al. 2016). Cryptolepine was shown to exert antiplasmodial activity against a multidrug-resistant (Kl) strain of *P. falciparum.* This study revealed that cryptolepine has a high IC_50_ value of 0.031 ± 0.0085 (SE) µg/mL, equivalent to 0.134 ± 0.037 µM. This suggests that cryptolepine has substantial potential as an antimalarial. Table 3 lists the antimalarial activities of cryptolepine, as well as its analogues.

### Anti-inflammatory activity

Inflammation plays an important role in maintaining homeostasis during acute inflammatory responses. Uncontrolled inflammation, however, can develop into chronic inflammation and result in a range of chronic illnesses, such as hepatitis, arthritis, and neurodegenerative disease. Cryptolepine exhibited anti-inflammatory activity activities both in vivo and in vitro. Furthermore, these activities have some molecular mechanisms that are still elusive and understudied. Cryptolepine inhibits the production of nitric oxide and the binding of DNA to NF-κB in vitro due to its anti-inflammatory properties. In vivo anti-inflammatory properties of cryptolepine were examined using a number of animal models of inflammation. Olajide et al. (2007) investigated that the synthetic cryptolepine-hydrochloride (2.5–10 µM) inhibited lipopolysaccharide (LPS)-induced nitric oxide production in murine macrophage cell line RAW 264.7. When cryptolepine observed the distinct steps of NF-κB activation, this excluded the possibility of an inhibitory effect on nuclear translocation of NF-κB caused by the alkaloid or degradation of IκB. A luciferase reporter gene assay revealed that cryptolepine inhibited the nuclear factor (NF)-κB, in human HEK 293 cells, which is crucial in the development of inflammatory and immune responses. Cryptolepine observed the separate steps of NF-κB activation and excluded an inhibitory activity on nuclear translocation of NF-κB by the alkaloid or degradation of IκB. These findings suggest that cryptolepine may suppress inflammation by inhibiting DNA binding of NF-κB activation and, consequently, transcription of proinflammatory proteins regulated by NF-κB (Olajide et al. 2007). The report by Olajide et al. (2009) documented the anti-inflammatory activity of cryptolepine and non-steroidal anti-inflammatory drug indomethacin; in comparison to those drugs, it was found that cryptolepine (10–40 mg/kg i.p.) had an anti-inflammatory activity dependent on the dose in acute carrageenan-induced rat paw edema. Oral administration of cryptolepine up to 40 mg/kg for four consecutive days inhibited lipopolysaccharide (LPS)-induced microvascular permeability and did not result in the formation of a gastric lesion in rats (Olajide et al. 2009). Olajide et al. (2013) reported the mechanisms of anti-inflammatory activities of the cryptolepine on lipopolysaccharide (LPS)-induced neuroinflammation in rat microglia. Microglial activation was induced by stimulation with LPS, and the effects of cryptolepine pre-treatment on microglial activation which was stimulated by LPS, PGE2/COX-2, microsomal prostaglandin E2 synthase, and nitric oxide/iNOS were examined. (Olajide et al. 2013). The results of this study revealed that cryptolepine significantly suppressed the levels of interleukin-6 (IL-6), LPS-induced production of tumor necrosis factor-alpha (TNFα), interleukin-1beta (IL-1β), PGE2, nitric oxide, protein, and mRNA levels of COX-2 and iNOS and LPS-induced p38 and MAPKAPK2 phosphorylation by targeting partially NF-κB signaling in the microglia (Olajide et al. 2013).

### Hepatoprotective activity

Hepatic fibrosis or liver fibrosis is a chronic scarring process that occurs in the liver due to prolonged liver injury caused by hepatitis virus, drugs, toxins, ethanol, and so on. Globally, it causes a substantial amount of morbidity and mortality. Cryptolepine derivative (HZ-6 h), induces liver fibrosis inhibition in TGF-β1-stimulated HSC-T6 cells by targeting the Shh pathway. In liver fibrosis, methyl-CpG-binding protein 2 (MeCP2) plays a central role, and silencing it could indirectly suppress collagen I and α-SMA (the marker of activated HSC) protein expression in TGF-β1-treated HSC-T6 cells (He et al. 2016). It was found that MeCP2 via up-regulating may contribute to hepatic fibrosis and then observed that HZ-6 h administered in a dose-dependent manner reduced the expression of MeCP2, α-SMA expression, and collagen I in HSC-T6 cells (He et al. 2016). Cryptolepine derivative (HZ-6 h) inhibited liver fibrosis in HSC-T6 cells by reducing MeCP2 expression. Cryptolepine has been tested for its biotransformation mechanisms and in vitro metabolism in human and rat hepatocytes. Cryptolepine disappeared rapidly between the plasma and various tissues except for the central nervous system; so, it can be concluded that the hepatic biliary tract is the main clearance pathway (Forkuo et al. 2017b).

### Antimicrobial activity

Cryptolepine has been found to be more effective against Gram-positive bacteria than Gram-negative bacteria in many studies. A number of researchers hypothesized that cryptolepine could lyse to *Staphylococcus aureus* cells. Sawer et al. (2005) observed antimicrobial activity of cryptolepine using the broth dilution method against the Gram-positive bacteria *S. aureus*. The result demonstrated that SEM photomicrographs after 3-, 6-, or 24-h treatment with 4X MIC (minimum inhibitory concentration), i.e., 20 µg ml^−1^ of cryptolepine, had a lytic effect on *S. aureus*. The staphylococcal cells’ surface morphological appearance was altered and the lytic effect coincided with low viable counts found in survival curves following cryptolepine administration (Sawer et al. 2005). Furthermore, cryptolepine has been evaluated as a possible antimycobacterial against a group of six species of fast-growing mycobacteria, such as *Mycobacterium smegmatis, M. fortuitum, M. abscessus, M. phlei,* and *M. aurum* (Gibbons et al. 2003). Assay results showed that this compound had a low MIC (16 μg/mL) which led to a further analysis against other mycobacteria namely, *M. phlei, M. aurum, M. smegmatis, M. bovis* BCG, and *M. abscessus* with MICs ranging between 2 and 32 μg/mL. The promising efficacy of the cryptolepine template against *M. tuberculosis* supported the ethnobotanical use of the extracts of *C. sanguinolenta* to treat infections (Gibbons et al. 2003). Cryptolepine was tested in vitro and in vivo for anti-bacterial activity against four *Babesia* species as well as *Theileria equi* and on the multiplication of *B*. *microti* in mice by Batiha et al. (2020). A fluorescence assay proved that the inhibitory effect existed. A toxicity assay using Madin–Darby bovine kidney (MDBK), mouse embryonic fibroblast (NIH/3T3), and human foreskin fibroblast (HFF) cells showed cryptolepine affected the viability of cells at a half-maximal inhibitory concentration (IC_50_) and half-maximum effective concentration (EC_50_) on *Babesia bovis*, *B*. *bigemina*, *B*. *divergens*, *B*. *caballi*, and *T*. *equi*. The results of this study showed that cryptolepine-atovaquone (AQ) and cryptolepine-DA combinations produced greater chemotherapeutic effects than cryptolepine alone. The cytotoxicity assessment on NIH/3T3, HFF, and MDBK cell lines exhibited that due to the high selectivity index of cryptolepine; it was more likely to influence the viability of *Babesia* and *Theileria* compared the host cells (Batiha et al. 2020).

### Antidiabetic activity

Diabetes mellitus is a pathologic condition characterized by a prolonged elevation of blood glucose due to insufficient insulin or insulin resistance. Diabetes has been associated with a number of complications leading to damage to the eyes, kidneys, and nerves and its most common symptoms are numbness, tingling, and pain. Hyperglycemia is a key factor in diabetic neuropathy development, and it not only increases the production of reactive oxygen metabolites but also inhibits antioxidative mechanisms through non-enzymatic glycosylation of antioxidant enzymes. According to Ameyaw et al. (2016), the antidiabetic activity of cryptolepine (10, 30, or 100 mg/kg) was evaluated against normal and alloxan-induced diabetic rats with altered fasting blood sugar (FBS), body weight, pain response, and semen quality with glibenclamide (10 mg/kg), or normal saline (2 ml/kg). Additionally, a comprehensive hematological profile, liver and kidney function tests, lipid profile, and liver, kidney, and pancreas histopathological investigations were completed as part of the cryptolepine treatment (Ameyaw et al. 2016). By using cryptolepine, it was possible to reduce fasting blood sugar levels and body weight, as well as decrease the latency to withdrawal from pain stimulus. by the treatment of cryptolepine. Cryptolepine was found to be dose-dependently (10–30 mg/kg) effective in regenerating long-term β-islet cells but could not repair degenerated liver and kidney tissue. It also reduced the quality of sperm in diabetic men (Ameyaw et al. 2016). The active ingredient cryptolepine suppresses symptoms of hyperglycemia, cold allodynia, weight loss, neuropathic pain, hyperlipidemia, and pancreatic β-islet cell damage associated with diabetes mellitus. According to Bierer et al. (1998b), hydroiodide, hydrochloride, and hydrotrifluoromethanesulfonate (hydrotriflate) salts of cryptolepine exhibited anti-hyperglycemic activity in rodent models of type II diabetes following assessment by a 3T3-L1 glucose transport assay (Bierer et al. 1998b). Table 4 summarizes the antidiabetic activities of cryptolepine and its derivatives.

### Anti-cholinesterase activity

Several studies have documented that some alkaloids like cryptolepine have always been a strong source of cholinesterase inhibitors. The cholinesterase (ChE) enzymes acetylcholinesterase (AChE) and butyrylcholinesterase (BChE) function to break down acetylcholine and some other choline esters that function as neurotransmitters, resulting in hindered neurotransmission (Nuthakki et al. 2019). The drugs functioning as cholinesterase inhibitors (CIs) have been found to reduce the cholinergic deficit in Alzheimer’s disease (AD) by restoring synaptic acetylcholine (ACh). Nuthakki et al. (2019) documented the anti-cholinesterase activity of cryptolepine and its 2-bromo derivative, which inhibit acetylcholinesterase and butyrylcholinesterase. The result of this study indicated that cryptolepine inhibits *Electrophorus electricus* acetylcholinesterase, recombinant human acetylcholinesterase, and the equine serum butyrylcholinesterase with IC_50_ values of 267, 485 and 699 nM, respectively (Nuthakki et al. 2019). The multi-targeted profile of cryptolepine, which inhibits both AChE and BChE enzymes, contributes to preventing Alzheimer’s disease.

## Conclusion

Cryptolepine and its analogues, derived from *C. sanguinolenta*, have attracted much attention in recent years, and researchers have been investigating these alkaloids to understand their pharmacological properties and mechanisms. Because of its positive pharmacological effects, cryptolepine could be used in the future as a molecule in integrative medicine, with major potential in preventing and reducing diseases and their manifestations. This review indicates that cryptolepine has a great potential in the treatment and prevention of several diseases such as malaria, intestinal disorders, cancer, urinary tract infections, rheumatism, and upper respiratory tract infections. Cryptolepine exhibits a wide range of pharmacological or biological properties including antimalarial, antimicrobial, anti-bacterial, anti-fungal, anti-hyperglycemic, anticancer, antidiabetic, anti-inflammatory, hepatoprotective, hypotensive, and antipyretic activities, presynaptic α-adreno receptor blocking action, antimuscarinic, and anti-cholinesterase activities. The anti-neurodegenerative effects of cryptolepine are mediated by its ability to inhibit enzymes such as acetylcholinesterase and butyrylcholinesterase. Cryptolepine has also been tested for its pharmacological activity against mice, rabbit, bacterial pathogens, fungal pathogens, and human cell lines. Several mechanisms of cryptolepine’s pharmacological activity have been addressed extensively in this study. In order to investigate the therapeutic applications of cryptolepine and its analogues, it is necessary to study their structure–activity relationships and elucidate the mechanism of their underlying effects, such as biosynthesis and signaling pathways, along with possible cross-talk by employing high-throughput technologies. There is still some research to be carried out on cryptolepine analogues so that they may be more suitable for human clinical trials in the future.

## Data Availability

Not applicable.

## Abbreviations

ACh: Acetylcholine
AChE: Acetylcholinesterase
AD: Alzheimer’s disease
BChE: Butyrylcholinesterase
ChE: Cholinesterase
CIs: Cholinesterase inhibitors
EC_50_: Half-maximum effective concentration
HFF: Human foreskin fibroblast
IC_50_: Half-maximal inhibitory concentration
IL-6: Interleukin-6
LPS: Lipopolysaccharide
MeCP2: Methyl-CpG-binding protein 2
NF: Nuclear factor
NOPR: NISCAIR Online Periodicals Repository
TGI: Tumor growth inhibition
TNFα: Tumor necrosis factor-alpha
